# Physical education and school sport in emerging nations: a comparison of Indonesia and Türkiye

**DOI:** 10.3389/fspor.2025.1582778

**Published:** 2025-06-09

**Authors:** Amung Ma’mun, Cem Tinaz, Anira Anira, Syarifatunnisa Syarifatunnisa, Ömer Onur Hertem, Agus Mahendra, Tite Juliantine

**Affiliations:** ^1^ School of Postgraduate Studies, Sport Education Study Program, Universitas Pendidikan Indonesia, Bandung, Indonesia; ^2^ Sport Studies, The Hague University of Applied Sciences, The Hague, Netherlands; ^3^ Middle Eastern Studies, University of Oslo, Oslo, Norway

**Keywords:** physical education, school sport, emerging nations, curriculum, out-of-school hours activities

## Abstract

**Introduction:**

This study examines physical education (PE) and school sport policies in Türkiye and Indonesia, focusing on their design, implementation, and institutional context. Both countries are emerging nations facing significant challenges in aligning curriculum objectives with available resources, infrastructure, and cultural attitudes toward PE.

**Methods:**

A qualitative research design was employed, involving semi-structured online interviews with thirty physical education teachers—fifteen from Türkiye and fifteen from Indonesia—working across different levels of public and private education. The interviews explored six key areas: curriculum objectives and applicability, school profiles, in-class sport activities, available resources, stakeholder attitudes, and extracurricular/club linkages.

**Results:**

Findings revealed common challenges in both countries, including limited infrastructure, insufficient numbers of qualified PE teachers in public schools, and a lack of systemic collaboration between schools and sports clubs. While Indonesian teachers viewed the curriculum as more adaptable, both countries struggle with resource limitations and implementation gaps.

**Discussion:**

The results highlight the need for government investment in school sports infrastructure, particularly gyms and multipurpose spaces, and for increased support for extracurricular and after-school sport programs. Formalizing partnerships between schools and local sports clubs is also essential to strengthening PE provision and long-term sports participation in both national contexts.

## Introduction

Government policies and funding for physical education (PE) programs are indispensable for fostering sport and community development. Many nations strengthen their PE curricula through legislation and administrative requirements. Interest and regulatory intervention in school sport and PE have been shaped as much by debates within the education policy area as by interests in several areas beyond education, such as social inclusion and community safety. In the UK, as with other Western countries, the promotion of sport and other forms of physical activity for young people has been one of the primary policy issues within the state's broader social inclusion agenda (1). The process of determining the national curriculum included two stages that were especially significant for PE: determining whether the subject would be included in the national curriculum; and if it were included, what status it would be given and what content it would cover (2). Hardman (3) pointed out that in Council of Europe member countries all students are required to participate in at least some form of PE and sports-related activities throughout their time in school.

This study aims to analyze and compare the state of PE and sport in Indonesia and Türkiye in a systematic manner. By examining the current state of PE and sports in both countries, we aim to gain a comprehensive understanding of the existing practices, policies, and frameworks that govern these areas. The comparative analysis aims to generate evidence-based insights that can inform policymakers in both countries. These insights will be valuable in formulating strategies and policies that enhance the quality and accessibility of PE and sports within the emerging nations context.

Whereas there has been considerable research and commentary around the ways Western countries organize and practice PE curriculum, relatively little is known about these phenomena in emerging nations. Here, cultural and socio-political contexts can impact what is done in the name of PE, as a curriculum practice in schools. The similarities that exist between these two countries make a comparison between them a meaningful endeavor. These main points of similarity can be categorized into three main categories: as developing economies, social practices associated with work and schooling, and the significant place that sport holds in their cultural identity. First, both countries are considered “emerging markets” according to the Morgan Stanley Capital Index in 2024. Emerging markets share several characteristics, such as robust economic growth, accessibility to foreign investors, a high per capita income, and a dependable regulatory framework—characteristics that both of these countries possess. In addition to the expansion of their economic capacities, both nations are deemed by many to have demographics that can sustain this growth. Sport in emerging nations plays a critical role in promoting social inclusion and economic development, yet challenges such as resource limitations and policy fragmentation continue to hinder its full potential (4). Indonesia is the fourth most populous country in the world, with approximately 275 million people, while Türkiye ranks 17th with 84 million. In addition, both countries have sizable youth population, which suggests that further growth is possible if the appropriate social and economic policies are implemented. Another parallel factor in both countries is that of religion, which is especially influential in Indonesia and partially Türkiye's respective national education policies. Officially, Indonesia has a system that can be described as a hybrid between secularism and an Islamic state. Türkiye, on the other hand, is officially a secular nation with a substantial majority of a Muslim society. In both countries, there are state-affiliated institutions that provide an Islamic education. Lastly, there are similarities between the two nations in terms of economic and social investments in sport. In recent years, both Türkiye and Indonesia have increased their investments in sports and their efforts to host major mega-events.

It is against this backdrop that this study aims to compare the landscape of PE programs in the two countries. The primary objective of this study is to comprehensively analyze the current landscape of Physical Education (PE) courses in schools within both countries, recognizing these courses as pivotal arenas for sports education. This investigation aims to uncover the critical factors influencing PE curriculum, delivery, and effectiveness, thereby providing a foundational understanding that can inform future policy and programmatic advancements in sports education. Our literature review has revealed that most of the studies on physical education and school sports in these two countries were written in Turkish and Indonesian. The intent of this paper is to address the gap in the international literature regarding the representation of physical education (PE) in Türkiye and Indonesia. A comprehensive understanding of PE and sports is imperative for the promotion of long-term social benefits, including improved public health, enhanced social cohesion, and greater overall well-being. Through the utilization of shared experiences and best practices, Indonesia and Türkiye can enhance their PE and sports systems, ensuring they function as catalysts for societal development, youth engagement, and inclusive community growth. In accordance with these purposes, this research aims to answer the following questions:

1. What are the primary objectives and educational goals of physical education curricula in Türkiye and Indonesia?
2. How are physical education classes structured and delivered across various educational levels and types of institutions in Türkiye and Indonesia?
3. What are the key challenges and critical issues faced by educators in conducting physical education classes in Türkiye and Indonesia?

## Literature review

Policy can be interpreted as a product of leadership (be it government or other types of organization), one of the choices to be used as a reference or guide in implementing activity programs for the public or members of the organization according to the goals and objectives they want to achieve (5). Governments produce policies in every area in which they have authority, such as public health, the economy, foreign relations, and sports. As sport is strongly connected with health, education, and social life, it is necessary for each state to have a sports policy and to execute this policy in coordination with other policies. Sport policy covers many different issues such as the principles and objectives of sport; the ways and methods of achieving these objectives; sport in education; the philosophy of organization and implementation of sport (6).

King (7) states that sports policy can embody a variety of causal claims, such as increasing participation, expanding the success of elite athletes, and reducing various social problems. Sports policies essentially focus on achieving instrumental results, such as delivering success to elite athletes, increasing the number of athletes, enabling talent identification and development paths, ensuring the sport is free from drugs and/or doping, and supporting sports organizations to recruit volunteers, coaches, officials, and administrators (8). As Ma'mun (9) explains, “national leadership has significant authority over the formulation of governmental policies, including those concerned with sports development.” Policy, he states, is closely related to the product of the persons or groups in positions of power who have produced the decision so that the decision in the form of the policy becomes a reference for the implementation of the program to achieve the expected goals of their leadership. Policy changes that occur in a short time can lead to a lack of cohesion in practice if policies are not in sync or overlap (10, 11).

In light of the intricacies inherent in the development and implementation of sports policy, it becomes imperative to undertake a thorough examination of the institutional frameworks that not only shape but also serve as the bedrock for the perpetuity of these policies. The conceptual framework provided by institutional theory offers a pivotal lens for the analysis of the formulation and execution of PE policies within the context of national education systems. This theoretical framework facilitates a nuanced understanding of how deeply entrenched norms and governance structures exert a profound influence on decision-making processes, thereby ensuring the long-term sustainability of these policies. Institutional theory is a sociological framework that explains how institutions—such as education systems, governments, and organizations—establish rules, norms, and practices that shape policies and behaviors within a given society. The theory posits that institutions evolve and persist over time, often resisting change due to embedded structures, traditions, and external pressures (12). Institutional theory provides a crucial framework for understanding how physical education (PE) policies are formulated, implemented, and maintained within national education systems. It posits that institutions, including educational frameworks, are shaped by broader socio-political and cultural forces that define their legitimacy, structure, and functionality (12). In the context of PE, institutional theory provides a valuable lens through which to understand the varied approaches taken by governments towards the integration of PE within national education. It helps elucidate the reasons behind the systematic integration of PE as a fundamental component of national education in some governments, while in others it is regarded as an ancillary component with minimal oversight or funding.

### School sport and physical education policies

Sport has become one of the most fundamental components of educational programs, although it is still sometimes described as a “game”. Sport has a further purpose than just being a simple game: Craig (13) explains that one of the most important social institutions in charge of this process is the education system. Extracurricular sports and athletic activities are frequently viewed as vital components of the education system. Sports in schools have been associated with teaching children a variety of socially beneficial values for more than a century (13–15). In addition, school sports have also been assigned, in some cases, with the task of combating student obesity (16–18).

Currently, the implementation of *school sport and physical education* policies can be categorized into three systems: the first one is the policies applied by the United States, the United Kingdom, and several other European countries that place a greater public role other than the state, in the sense that sports are already in the industrial area, where the public is very dominant, except those that are inherently the responsibility of the state, such as Physical Education and School Sport (PESS) (19). Secondly, the policies applied by China are very different since all the ins and outs relating to the development of the state/government sports or the presence of the state/government are always the most significant part of its contribution (20). The third and last one is the policy that are between these first two systems. For instance, in Indonesia (9), where the presence of the state becomes the focus of hope accompanied by efforts to develop the participation of the public at large in accordance with Law No. 3/2005 (21) on the National System of Sports. Also, Türkiye is among the countries in this category along with Indonesia. Although the state is at the center of the promotion of sport and physical education and it has all the regulation and control mechanisms in terms of sports and physical education, there is a diversity in terms of implementation between different actors such as schools, federations, clubs, within the limits of their capabilities.

The UK led the way, especially in the early 2000s, in terms of policy and development of school sports for the European Region and managed to provide the foundation for the importance of PESS. As mentioned above, PESS is not the only existing PE model, yet it is an exemplary model for many countries trying to improve their sports and educational policies, such as Indonesia. PESS has four characteristics in its application, namely: PE, out-of-school hours activities (OSHA), talent development, and club links as sports development, as well as in the community as recreational activities and achievements (22). PESS is very substantial because it concerns the formation of young people in various domains in the expected direction, so that they will be the people who contribute and take responsibility as a lifestyle.

Especially since the beginning of the twentieth century, the UK has made unprecedented central government policy commitments that invest in PE and sports. The reasons for this investment lie in public health conditions: rates of obesity with a predicted health care cost of £2 billion per year; sedentary lifestyles; and the high drop-out rates of youth from sports (23). The existence of PESS in the structure of school activities in the UK is not only seriously discussed among educators or subject teachers, as is the case in Indonesia, but political dialogue grows and develops very constructively between experts in the field of PE and sports with politicians, especially regarding policy discourse (24).

By contrast, in the United States, school-based physical education (SBPE) is not regulated at the national level. There is only the recommendation established by the National Association for Sport and Physical Education (NASPE), Guidelines for Quality Physical Education (25), regarding the amount of SBPE instructional time. The guidelines stipulate that children have the opportunity to learn, which necessitates at least 150 minutes per week of SBPE instruction at the elementary school (ES) level and 225 minutes per week of SBPE instruction at the middle school (MS) and high school (HS) levels.

The lack of a national law regarding SBPE invites ambiguity in the definition of SBPE itself. Despite the existence of guidelines for quality PE, there are still numerous states that do not adhere to these guidelines. Most state SBPE laws fail to adequately define the content that must be taught in schools and do not expressly require the amount of SBPE teaching time that complies with the Guidelines at all levels (26). Descriptive statistics indicated that, at the elementary school level, 41 states (82%) had a mandate for SBPE instruction, and only six of these states (12%) followed the guidelines of 150 minutes of SBPE instruction per week. At the MS level, 37 states (74%) had a mandate for SBPE instruction and two (4%) adhered to the guidelines of 225 minutes of SBPE per week. Forty-one states (82%) had a mandate for SBPE instruction at the HS level, but none had a mandate adhering to the Guidelines.

This situation reveals that having multiple policies does not provide absolute clarity on the implementation of PE. On the other hand, the absence of policies related to PE is also a problem. Such a scenario leaves administration of each state with no direction on how to implement PE in schools. Outside of these two categories which were presented in the previous section, Türkiye and Indonesia, as a part of a third category, have different systems of physical education and sport policy that can be described as hybrid policies. In Türkiye, curricula are comprehensive but unrealistic, as resource shortages prevent full implementation (27). Investment disparities between private and public schools exacerbate the issue. In Indonesia, teachers assess the curriculum as more adaptable than their Turkish counterparts, yet 50% of Indonesian teachers report that facility shortages prevent its proper implementation (28).

### Sport and physical education policies in Indonesia and Türkiye

In this section, we provide an overview of the histories of sport and physical education in the two countries, based on official documents such as curriculum, statistics of national institutions and existing literature.

Indonesia has undergone many curriculum changes, including the 1947, 1952, 1964, 1968, 1975, 1984, 1994, 2004, 2006 curriculum, and most recently is Curriculum 2013 (29). Curriculum-2013 (C-13) has four aspects of assessment, namely aspects of knowledge, aspects of skills, aspects of attitudes, and behavior. In the structure of the 2013 Curriculum, the subjects of Physical Education, Sports and Health (PESH) have content to develop movement competencies and healthy lifestyles and give color to the nation's character education. Learning PESH with local wisdom will give appreciation to multiculturalism, namely getting to know traditional games and sports that are rooted in Indonesian ethnic culture and can contribute to character building. Learning is carried out with a scientific approach, namely observing, questioning, association, experimenting, creating, and communicating.

In Indonesia, every citizen aged seven to fifteen years is required to attend basic education (9 years from primary to secondary schools) (30). The funding is provided by the government. PESH is one of the compulsory subjects from Primary School to High School in Indonesia. Primary school is held for six years with a student age of seven years. After graduating from primary school, students then continue to secondary schools for three years, and then to high school for another three years. There are no significant differences between types of school in Indonesia regarding the time allocation in PESH for public, private, religious, vocational, and boarding schools. All the students have the same amount of time and the same curriculum for all types of schools according to the availability of school facilities. Currently, PE policy has two main characteristics, namely: sports and health, and extracurricular subjects. In the past, extracurricular sports activities were often conducted by students but not permanently institutionalized, as was done by the UK by including PESS as an integral part of its curriculum. Recently, sports activities outside of school hours have been decreasing due to public spaces having become more and more limited. This is because the schools generally do not have adequate facilities and open spaces.

Sport in Türkiye has received greater attention as a government policy concern over the last decade, one which is characterized by direct government involvement in every sporting field and at every level (31). Türkiye's sports policy adopts the priority of school-based and grassroots youth sports development, and this is how it is planned to continue. Reforms were made in the field of sport education, and as a part of this, the PE curriculum was last renewed in 2017. Although many changes have been made to the curriculum in recent years, according to most academic studies, the most important obstacle to physical education is not the curriculum as such, but rather material barriers such as lack of facilities or equipment (27, 32). While much of the literature in Türkiye has focused on public schools as an example and as a result issues such as lack of facilities or inadequate equipment have come to the forefront, the inadequacy of the training received by teachers, the lack of a supportive attitude of the school administration, lack of the physical activity of the teachers and the curriculum being prepared with unrealistic expectations are also common problems mentioned in the studies (33–36).

From a broader perspective, there have been far more comprehensive changes in recent years, not only in the physical education curriculum but also in the education system. In 2012, primary education was reduced from 5 to 4 years and secondary education was increased from 3 to 4 years. With this change, religious secondary school, *İmam Hatip Ortaokulları*, which had been abolished in 1997, were reintroduced into the education system. Currently, compulsory education in Türkiye is 12 years, with 4 years of primary education, 4 years of secondary education and 4 years of high school. In high schools that give education in a foreign language, depending on the language level of the student, one year of language preparatory education can be also compulsory. In the level of primary education, private primary schools and public primary schools use the same curriculum. According to this curriculum, “Physical Education and Game” class is compulsory for the first 3 years, 5 hours a week. In 4th grade, its duration is reduced to two hours per week. From the 5th grade of primary school until the end of high school, the course is called physical education and sports (37).

At the secondary school level, public schools are divided into two categories: general secondary schools and religious secondary schools. One of the differences between these two school types is physical education class, which is compulsory and two hours a week in general secondary schools, is optional and two hours per week in the first year and only one hour for the second, third and fourth year in religious secondary schools. Moreover, “Basic Religious Knowledge” classes, which are elective courses in general secondary schools, are compulsory in religious secondary schools.

At the high school level, diversity in terms of school type increases. In total, there are 10 types of high schools in Türkiye, but 4 types stand out as having a very large majority. These are Anatolian High School “Anadolu Lisesi”, Religious High School “İmam Hatip Lisesi”, Vocational High School “Meslek Lisesi”, and High school of Science “Fen Lisesi”. The first main difference between these schools is that religious high schools give education to the boys and girls separately, while it is mixed in the other high schools. The differences between the focus areas of these high schools are also reflected in the physical education class. Problems such as the curriculum and lack of facilities will be analyzed in more detail in the next section of the study through interviews with teachers.

## Methodology

A case study approach was chosen as the research methodology of this study, which was built around curriculum document analyses corroborated by semi-structured in-depth interviews with PE teachers from both countries.

In the first phase of the research, we focused on the primary sources, especially the national curriculums of the PE courses in Indonesia and Türkiye, and official documents of each country's Ministry of National Education and Ministry of Sport and Youth. Türkiye has three distinct PE curriculum for primary, secondary, and high school education, whereas Indonesia has a single curriculum for all educational levels. The comparative document analysis entailed a systematic review and thematic coding of national education and curriculum policy documents from Indonesia and Türkiye. The Indonesian documents analyzed included *Curriculum 2013*, *Law No. 20 of 2003 on the National Education System*, and *Government Regulation No. 57 of 2021 concerning National Education Standards*. For Türkiye, the analysis encompassed the national *Physical Education and Play Course Curriculum* for primary schools, as well as the *Physical Education and Sports Course Curriculum* for middle and high schools, all published by the Ministry of National Education (MEB) in 2018. Additionally, the curriculum guidance document published by the Ministry of National Education in 2021 and the 2024–2028 Strategic Plan, also released by the same institution in early 2024, were examined (53–56). These documents were analyzed to identify, categorize, and compare key themes and policies that shape sport development in both countries.

In the second stage, we conducted fieldwork to analyze how realistic and applicable the curricula of the PE courses in these countries are and how PE lessons are practiced in different educational institutions. At this stage, semi-structured interviews were conducted with a total of 30 PE teachers actively working in Baldung, Indonesia and Istanbul, Türkiye—with 15 teachers from each country respectively. For the Indonesian part, five of these teachers are employed in primary schools, four of them in secondary schools, and six of them in high schools. Of the five teachers working in primary schools, two of them work in private schools and three in public schools, while all four teachers working in secondary schools work in public schools. At the high school level, four teachers work in private schools and two teachers work in public schools. In total, 9 teachers work in public schools, and the remaining 6 are in private schools. In Türkiye also, a total of 15 teachers were interviewed. Six of these teachers work in secondary schools, and nine of them are in high schools. Of the six teachers working in secondary schools, three of them work in private schools and three work in public schools. Among the high school teachers, one of them works at a private school and the remaining eight work at public schools. In total, 4 teachers work in private schools and 11 in public schools. The selection of teachers working at different types and levels was aimed at obtaining a result that could reflect the overall situation in the country. In order to preserve the anonymity of the teachers who participated in the interviews, the teachers from Türkiye were coded as Turkish Teacher (TT) 1–15 and the teachers from Indonesia as Indonesian Teacher (IT) 1–15.

All interviews were conducted online, recorded and transcribed to ensure accuracy. The transcribed data were then translated into English to facilitate collaborative analysis. MAXQDA qualitative data analysis software was used to systematically interpret the findings. An open coding approach—including both inductive and deductive methods—was used to identify recurring themes and extract key findings related to PE and sport. To enable structured comparison, an analysis template was developed that categorized teachers’ responses into six critical dimensions: curriculum objectives and applicability, school profiles, in-class sports activities, availability of PE resources and facilities, attitudes of school administrators and students, and extracurricular activities, including links with sports clubs. This structured approach allowed for a nuanced examination of patterns, challenges and good practice in the two national contexts. The data collected at this stage aims to determine how state-level decisions regarding sports policy and PE classes reflect reality. The responses from the 30 participants enabled comparisons between countries and between private and public schools within the same country.

## Findings

The findings of a comparative analysis of the data obtained from the examination of the Indonesian and Turkish curricula and the semi-structured interviews are presented under six headings. These are the profiles of the teachers and their schools, the in-class sport activities, the available resources for PE classes in school sport activities, the attitudes of the directors and students, the extracurricular activities and connections to sports clubs, and the curriculum's applicability.

### Objectives and applicability of curriculum

In terms of the objectives of the learning curriculum, there is no significant difference between Indonesia and Türkiye. At the elementary school level, both have the same goal of helping students to understand the basics of movements and their combination. At the secondary school level, students are expected to be able to understand and explain concepts and skills in games and sports. Furthermore, at the high school level, students should be able to practice and analyze the importance of physical activity and maintaining physical fitness. Aside from these goals, both Indonesia and Türkiye also have broader social aims such as promoting honest and caring behavior, discipline, fair play, responsibility, sports ethics, and tolerance.

Regarding the applicability of the PE curriculum, Indonesian participants responded slightly differently than their Turkish counterparts—a difference that can be explained by the difference in curricula and also by teachers’ perspectives on curricula. Although the primary concern from both countries was the lack of adequate facilities and equipment, unlike in Türkiye, a group of Indonesian participants stated that they teach their course in accordance with the curriculum. Nearly half of the participants share this view on the curriculum and this number is considerably higher than that of teachers in Türkiye. In Indonesia, important topics such as weekly lesson time, student rights and responsibilities have been placed at the heart of the curriculum, and the teacher has been given a great deal of autonomy in implementing the lessons. Consequently, Indonesian teachers can assert that they are teaching this course according to the curriculum if they adhere to the weekly lesson time standard and respect students’ rights.

Regarding the applicability of the curriculum, IT14 who works in a public primary school, stated:

“The PE learning program carried out refers to the curriculum, and its application is adapted to the situation and conditions at school.”

On the other hand, the other half of the Indonesian teachers claimed that the implementation of the curriculum in PE classes is limited by the school's current infrastructure. According to most teachers, the quality and quantity of facilities and infrastructure for PE classes play a crucial role in achieving the curriculum's goals and meeting the expected standards. IT8, working in a public primary school, pointed out the difficulty of implementing the curriculum:

“We find it difficult to implement the curriculum recommended by the government because the facilities and infrastructure at the school are very limited. We only do PE lessons by utilizing existing facilities.”

In the Turkish context meanwhile, even though the curriculum is written quite comprehensively and effectively, almost all teachers asserted that it is impossible for them to follow it in its entirety with the resources they have. As previously mentioned, there are still many schools without gyms, balls, rackets, etc. that it is impossible for teachers at these schools to carry out some of the activities outlined in the curriculum. According to the teachers, they take individual initiative and teach the lesson as best they can in such circumstances. Although the national curriculum of Türkiye appears to be very successful in terms of scope and vision at first glance, it can be argued that it proceeds from an unrealistic perspective. In the end, the most challenging aspect of implementing the curriculum is related to financial constraints that limit access to sports facilities and equipment. TT4, who works in a public general secondary school, made a remark on this issue:

“The sports infrastructure of the schools is not suitable for realizing the curriculum. No sports equipment is provided to schools in any way. We buy sports equipment by collecting money from the school's family union. The subjects in the curriculum may sound good, but they are very difficult to implement.”

### Profile of the schools

The first striking detail in the participants’ answers from Türkiye is the difference in the number of students per PE teacher between private and public schools. While there is one teacher per 103 students on average in the private schools examined in the research, this number rises to 400 in state schools. Such a statistic clearly suggests that the workload of teachers working in public schools is almost four times heavier than that of the average private school teacher. As a result of such a demanding setup, the time a teacher who works in a public school can spare for a student is four times shorter compared to private schools. This difference not only increases the stress on public school teachers, but also leaves them with less time to develop themselves in their field. This situation is one of the first reflections of the difference between private and public schools in Türkiye, as we will frequently mention in other sections. TT4, who works in a public general secondary school, reflected on this situation:

“It is almost impossible to communicate effectively with students and also discipline them when we have quite limited time and more than a hundred students to take care of.”

In addition to this, TT10, working in an Anatolian high school, mentioned:

“The PE teacher is an important factor in establishing order and discipline in the school, but it is challenging to achieve this under these conditions.”

In Indonesia on the other hand, the students-per-teacher gap between private and public schools is nowhere near as dramatic, with 223 students per teacher in private schools and 258 in public schools. According to these statistics, unlike in Türkiye, there is no difference in terms of this particular point in Indonesian schools between the private and public sectors.

Supplementary Table 1 provides an overview of school types and duration of the PE lectures both in Indonesia and Türkiye.

### In-class sport activities

The national PE curricula of the two countries and the data provided by the participants indicate that, with only two exceptions, each type of school studied devotes at least two hours per week to PE. The vocational high schools in Türkiye represent these two exceptions, where students do have at least two hours of PE classes per week until their final year, when that becomes limited to one hour. In both countries, most students participate in PE classes for at least two hours per week throughout their entire schooling period.

In both countries, the course's activities are nearly identical, primarily consisting of football, basketball, volleyball, and swimming, as these sports are the most popular and easiest to practice with the available resources and facilities. In addition, traditional and local sports such as martial arts are occasionally included in Indonesia. In Türkiye, private schools appear to offer a wider variety of in-class sports activities than public schools due to their greater financial and physical resources, whereas in Indonesia, extracurricular activities are similar in both private and public schools.

### Available resources for PE and in school sport activities

Almost all participants, regardless of the country or type of school in which they teach, agreed that the availability and quality of the facilities and sports equipment are the most crucial factors for delivering an effective class. The choice of in-class activities is also directly related to these factors. First, only six of the fifteen participants in Türkiye indicated that their school has a gym suitable for the PE class, with nine teachers reporting that they had conducted lessons in the schoolyard. One striking difference between the public and private sectors was that all of the private schools examined in the study have at least one gym, whereas only two of the public schools do. In almost every question, differences between private and public schools in Türkiye are evident. However, only one of the Indonesian teachers has a gym in his school. This is due to the fact that the sports facilities in that particular school are shared with the facilities of the Indonesian Air Force, while the remaining 14 teachers we spoke to conduct their classes in the schoolyard or in another open area.

The lack of a gym has a direct impact on how and when the lesson can be conducted. When the weather is unsuitable for sports, particularly during the fall and winter months, it is frequently impossible to hold the lesson. In this instance, the teachers stated that they try to transform the lesson into theoretical education or engage the students with indoor games. However, because in some regions such weather conditions can endure for quite a long time, some students do not engage in any physical activity for nearly half the year, which is incompatible with the course's objectives. TT9, who works in a public Anatolian high school, made a remark about this issue:

“As we do not have a gym, it is not really possible to do any sports activities from November until March, mostly because of the weather conditions.”

Similarly, IT5, working in a private high school, on the same issue:

“We have facilities, but they are far from perfect—only one basketball court—and sometimes they are used in conjunction with PE lessons taught by other teachers. All PE learning activities are carried out in the schoolyard.”

In addition to the lack of school gyms, the availability and quality of the equipment that can be used in the lessons play a crucial role in the effectiveness of the lessons, and there are clear differences between schools in this regard. The curricula of the two countries include a wide variety of sports, each of which requires its own specific equipment, which teachers often find themselves struggling to provide. IT11, who works in a private high school, commented on this issue:

“We have facilities, but the problem is that sometimes the field is used for parking because schools do not have parking spaces, and for the equipment of learning tools, we usually modify it.”

The teachers, who are unable to afford new equipment, stated that they had used the same materials for years and even purchased sports equipment for the school with money from their own pockets on occasion. Inadequate or insufficient materials decrease student interest in the lesson and diminish the effectiveness of the lesson's instruction. Except for private schools in Türkiye, this is what was reported to us regarding all the schools where the participants are employed. Almost all the teachers claimed that they had to modify their equipment to support PE learning.

### Attitudes of the directors and students

In both countries and across all types of schools, most students appear to be interested and engaged in PE. Considering the teachers’ statements, it is possible to say that students are especially eager to play popular sports, even though the opportunities are insufficient. TT6, teacher in a private secondary school, remarked on this:

“In general, if the children want to play sports, we support them. Mostly boys are particularly interested in soccer and girls in volleyball.”

On the other hand, there were also some examples of schools where the course was not given much importance by students, or more so, by their parents. The most important reason for this is the families’ expectations of academic success. In this context, among students in the final year of vocational high schools or some other high schools where PE is an elective course, it is occasionally observed that a subset of students are hesitant to enroll due to a lack of facilities and equipment or the pressure to perform well in other classes. Especially for students who are preparing for the university entrance exam—and for their families—the PE lesson can appear to be a waste of time. TT9, who works in a public Anatolian high school in Türkiye, offered some insights on this.

“Due to the pressure from their parents or school directorate, academic success is always the priority for the children. Voluntarily or not, some of them take a step back when it comes to sports.”

Another potential problem may occur amongst the students in the Religious High Schools. As the teachers we interviewed here pointed out, sometimes the participation of female students in class can be restricted by their families. The main reason for this is that families believe that the clothes worn, or actions taken during class are religiously inappropriate.

In terms of administrators in both countries, they are generally seen to have a supportive attitude, although the level of support varies between schools. Some school administrators allocated funds for physical education classes from the annual budgets sent to their schools by the Ministry of National Education. They also established teams in various sports and contributed to the school's participation in regional and national sports competitions. TT 8, who works in a private secondary school in Türkiye, made this statement regarding the school administration's approach to their PE course.

“Our school supports us in participating in personal professional development sessions. At the same time, our school obliges us to receive training on subjects such as psychology, etc. We are a small school that supports sports very much.”

Although no participant reported that their administration had a directly negative attitude toward PE classes, a few participants stated that PE was occasionally treated less seriously than other courses by the school directors. This can lead to both the course budget being restricted and students realizing this and also not taking the course seriously. IT3, a teacher in a private primary school, made a remark on this issue.

“The school management team works by existing plans and regulations. The service for improving the quality of learning in PE and sports has not become an important priority.”

TT2, who works in a vocational high school, expressed a similar sentiment.

“There is only one expectation from PE lessons in high schools: teachers should be with the students and help them to use their energy. Their only concern is that PE classes should be done, and students should not create problems for others.”

These behaviors and expectations of the administration may lead students to see the PE course as a leisure activity rather than a valid subject. According to teachers who have experienced this, it also causes problems in maintaining discipline and order in the school in the long run.

### Extracurricular activities and connections with sports clubs

In Indonesia, 12 out of the 15 schools studied were found to engage in extracurricular sports activities, such as establishing school teams in football, basketball, volleyball, etc. to organize regular practices and participate in tournaments at different levels. In Türkiye, the number of schools with extracurricular sports activities is 11 out of 15. Given the facilities and equipment available to schools, especially public schools which do not have access to a gymnasium and most of the necessary equipment, these figures are quite high. Here, the driving forces would seem to be the students’ desire, the teachers’ effort, and the teachers’ network. Nevertheless, just as with in-class activities, school facilities play an important role in extracurricular activities. Even if students and PE teachers are motivated to participate in a particular sport or establish a team in different branches, this motivation can often lead to very little if the school does not have the necessary facilities.

In both nations, extracurricular activities, such as school team training and courses taught by the teachers in their area of expertise, appear to be within the capabilities of the schools represented. With two exceptions in Türkiye, the relationship between sports clubs and schools has remained either in cooperation with local youth clubs or within the teacher's personal network. Two of the private schools featured here have their own amateur sports clubs, and PE teachers refer talented students to these organizations. Aside from these two schools, in both countries the relationships between schools and professional clubs are either very tenuous, nonexistent, or established solely through the personal connections of teachers.

TT9, a teacher in an Anatolian high school, stated:

“From time to time I recommend talented students to sports clubs, but it's not like a link between school and club, it's more of a personal network.”

IT15, who is a private high school teacher, argued that the school's sporting activities and PE classes were not linked to each other and stated:

“We are active in sports organizations. However, it has nothing to do with PE at school.”

On the other hand, TT2, working in a vocational high school, looked at the issue from a different angle:

“There used to be good cooperation between youth teams, but it is not so established anymore. We hope that [it] can be reestablished like it was before by the school inviting and re-embracing the youth team to support school sports activities.”

## Summary and discussion

This study critically evaluates PE policies in Türkiye and Indonesia, focusing on policy implementation, resource allocation, and their broader role in sports development and participation. A compelling rationale for comparing Türkiye and Indonesia lies in their shared classification as emerging economies with parallel demographic and socio-political structures. Both countries possess large, youthful populations and are navigating educational reform amid rapid urbanization and economic development. Additionally, each demonstrates strong governmental involvement in PE and sport policy yet faces persistent gaps between policy formulation and implementation. Despite these similarities, the nations also diverge in administrative structures, religious influences, and educational decentralization, offering a rich comparative framework. This duality of resemblance and distinction enables a deeper exploration of how institutional factors shape PE outcomes in emerging nation contexts, contributing to the under-researched global discourse on PE and school sport outside Western paradigms (4, 9).

Supplementary Table 2 presents a comparative summary of key aspects of Physical Education and School Sport implementation in Türkiye and Indonesia. This comparison highlights the structural, institutional, and cultural factors shaping the delivery of school sports in both national contexts.

Both Türkiye and Indonesia have well-defined PE policies, with curricula that emphasize fundamental movement skills, values of sportsmanship, and long-term participation in physical activity (3). However, there is a significant gap between policy aspirations and practical implementation, attributed to resource shortages and systemic inefficiencies. PE is institutionally recognized in both countries, yet it is not prioritized in practice due to financial constraints and competing educational demands.

A critical finding is that PE implementation is directly tied to resource allocation, which is neither proportional nor equitable. Public choice theory suggests that governments distribute resources based on political and economic priorities (38). In both countries, elite sports receive disproportionately higher funding than school-based PE programs. In Turkey, the gap between the public and private sectors is severe, with private schools offering significantly more opportunities for physical education than public schools. Unlike many developed countries, where robust school sports programmes actively encourage sport participation, Türkiye's education system does not yet have a comprehensive sports development framework (39). Public school PE teachers manage four times more students than their private counterparts, and only two out of eleven public schools have indoor sports facilities, compared to 100% of private schools in the study. In Indonesia, insufficient funding has been identified as a key challenge affecting both public and private schools. Concurrently, the limited availability of shared sports facilities has been found to impede access to structured physical activity sessions, thereby contradicting the observations reported by Fitri et al. (40).

A major limitation in both nations is the lack of formal school-club partnerships, despite their success in European sports development models (41, 42). While school-club collaborations have been successfully institutionalized in Europe, neither Türkiye nor Indonesia has systematic policies promoting structured partnerships. Currently, existing partnerships are informal and teacher-driven rather than government-supported. Successful European models emphasize structured collaborations, enabling early talent identification (43).

Beyond resource shortages, cultural perceptions significantly influence PE participation. Institutional theory posits that education policies reflect broader societal norms and values, shaping student engagement in PE (44, 45). A fundamental principle of Institutional Theory is the delineation between formal rules (legislative policies, curricula, and funding mechanisms) and informal norms (cultural perceptions, social attitudes, and community engagement) that influence institutional practices (46, 47). In the context of Türkiye and Indonesia, the existence of formal PE policies is acknowledged, yet their implementation is frequently impeded by informal institutional deficiencies, including a paucity of prioritisation, inadequate infrastructure, and competing national educational objectives. In Türkiye, female participation is lower in religious schools due to social norms restricting sports participation. Some families prioritize academic success over PE, seeing it as an expendable subject (48). In Indonesia, academic pressures sometimes result in PE being deprioritized in schools. While gender-related restrictions are less pronounced than in Türkiye, sports participation is undervalued (49).

To bridge the gap between policy and practice, several strategic interventions are necessary. Governments should conduct real-time curriculum reviews to ensure policies are achievable with existing infrastructure. Minimum PE facility standards should be established across all schools to improve accessibility (50). Public PE budgets must be increased, particularly in low-income public schools, while corporate sponsorships should be encouraged to support school PE programs (51). Institutionalizing school-club partnerships is critical; national frameworks linking schools with local clubs and federations should be developed, and financial incentives should be provided for clubs to support youth sports development (57). Overcoming cultural barriers to participation requires implementing culturally responsive PE curricula and introducing parental education initiatives on PE's long-term benefits (52).

Türkiye and Indonesia recognize PE's value, but systemic barriers prevent full implementation. Aligning curricula with available resources, increasing public school funding for PE, institutionalizing school-club collaborations, and addressing cultural barriers to PE participation are essential steps toward a more equitable PE system. Future research should explore how PE policies can be adapted across diverse socio-economic contexts to ensure effective implementation.

## Data Availability

The original contributions presented in the study are included in the article/Supplementary Material, further inquiries can be directed to the corresponding author.
