# Supplementary material for: Physical education and school sport in emerging nations: a comparison of Indonesia and Türkiye
Source: Front Sports Act Living. 2025 Jun 9;7:1582778. doi: 10.3389/fspor.2025.1582778 (PMC12184766; doi:10.3389/fspor.2025.1582778)
Supplement: Supplementary file 1 [file Table1.docx]

| Table 1. Characteristics of Curriculum PE in Indonesia and Turkey | | |
| --- | --- | --- |
| **Variable** | **Indonesia** | **Turkey** |
| **Total years in school** |  |  |
| Primary School (years) | 6 | 4 |
| Secondary School (years) | 3 | 4 |
| High School (years) | 3 | 4 |
| **Compulsory PE lectures hours per week** |  |  |
| Primary School | 4 x 35 minutes | 5 x 40 minutes in first 3 grades  2 x 40 minutes in the 4th grade |
| Secondary School | 3 x 40 minutes | 2 x 40 minutes |
| High School | 3 x 35 minutes | 2 x 40 minutes |
| **Main Goals of PE lesson** |  |  |
| Primary School | 1. Understanding the variations and combinations of basic locomotor, non-locomotor, and manipulative movements with reasonable control in big ball games, small ball, self-defense, and gymnastics activities 2. Understanding physical fitness exercises and measuring personal physical fitness levels in a simple way 3. "Show honest, disciplined, responsible, polite, caring, and confident behavior in interacting with family, friends, teachers, and neighbors and love the homeland" | 1. Students understand the basic and combined movement skills effectively and confidently in simple rule games and physical activities. 2. Willingly participating in games and physical activities regularly to stay healthy and improve physical fitness, recognizing and practicing traditional dances and dances of our culture and other cultures, and participating in festivals, celebrations, and ceremonies. 3. Students willingly develop self-awareness, individual responsibility, self-confidence, and time management skills through games and physical activities, communication, leadership, cooperation, and respect for differences. |
| Secondary School | 1. Understand the variations and combinations of specific movements in various big ball games, small ball games, self-defense, and floor gymnastic activities 2. Understand the physical fitness development program related to health and skills in a simple way 3. "Show honest behavior, discipline, responsibility, caring (tolerance, mutual cooperation), polite, and confident in interacting effectively with the social and natural environment within the reach of the association and its existence" | 1. Active and healthy life. Regular physical activities, terms and principles of physical activities and cultural values. 2. Explaining the basic movement concepts used in games and activities and practicing these sports in accordance with rules. 3. Explaining the importance of cooperation, respect and fair play concepts in games and activities. |
| High School | 1. Designing an exercise program to improve the degree of physical fitness related to personal health and skills 2. Analyzing the skills of two styles of swimming for self-rescue skills, and emergency rescue actions in the water using assistive devices 3. "Show honest, disciplined, responsible, caring behavior (mutual cooperation, cooperation, tolerance, peace), polite, responsive and pro-active as part of the solution to various problems in interacting effectively with the social and natural environment and placing oneself as a reflection nation in the association of the world”. | 1. Practicing the warm-up and basic movements specific to the selected sport and explaining the rules of this sport. 2. Explaining the importance of doing regular physical activities to improve physical fitness and encouraging students to continue doing physical activities during their life. 3. Explaining both the national and global history and importance of sport and raising awareness among students about the spirit of sports ethics and fair play. |
