# Supplementary material for: Physical education and school sport in emerging nations: a comparison of Indonesia and Türkiye
Source: Front Sports Act Living. 2025 Jun 9;7:1582778. doi: 10.3389/fspor.2025.1582778 (PMC12184766; doi:10.3389/fspor.2025.1582778)
Supplement: Supplementary file 2 [file Table2.docx]

| Table 2. Comparative Overview of PE and School Sport in Türkiye and Indonesia | | |
| --- | --- | --- |
| **Theme** | **Türkiye** | **Indonesia** |
| **Curriculum Objectives** | Broad and ambitious objectives outlined; challenges in aligning with school realities. | Curriculum objectives generally considered achievable, with some flexibility in interpretation. |
| **Curriculum Applicability** | Difficult to implement due to lack of resources and institutional support. | More adaptable in practice but constrained by infrastructural limitations. |
| **School Characteristics** | Marked disparity in provision between public and private schools. | Less variation between public and private institutions. |
| **In-Class Activities** | Limited activity diversity, especially in under-resourced public schools. | More consistent activity offerings across schools, but facilities often shared or improvised. |
| **PE Resources and Infrastructure** | Widespread gaps in access to sports facilities, especially in public schools. | General lack of dedicated facilities in both public and private schools. |
| **Extracurricular and Club Links** | Informal and largely dependent on individual teacher initiative. | Similar reliance on informal networks; limited institutional partnerships. |
